# Gender and Cooperation in Children: Experiments in Colombia and Sweden

**DOI:** 10.1371/journal.pone.0090923

**Published:** 2014-03-10

**Authors:** Juan-Camilo Cárdenas, Anna Dreber, Emma von Essen, Eva Ranehill

**Affiliations:** 1 Department of Economics and CEDE, Universidad de los Andes, Bogotá, Colombia; 2 Department of Economics, Stockholm School of Economics, Stockholm, Sweden; 3 Department of Economics and Business, Aarhus University, Aarhus, Denmark; 4 TrygFonden's Centre for Child Research, Aarhus University, Aarhus, Denmark; 5 Department of Economics, University of Zürich, Zürich, Switzerland; University of Zaragoza, Spain

## Abstract

In this article we compare cooperation among Colombian and Swedish children aged 9–12. We illustrate the dynamics of the prisoner's dilemma in a new task that is easily understood by children and performed during a physical education class. We find no robust evidence of a difference in cooperation between Colombia and Sweden overall. However, Colombian girls cooperate less than Swedish girls. We also find indications that girls in Colombia are less cooperative than boys. Finally, there is also a tendency for children to be more cooperative with boys than with girls on average.

## Introduction

The possibility to overcome socially inefficient economic outcomes through cooperation plays an important role in many everyday situations such as those concerning the provision of public goods, or the use of common-pool resources. Cooperation varies substantially among individuals and across contexts [1], [2], [3], yet relatively little is known about the formation of preferences for cooperation across the life cycle and how it varies between cultures. Understanding the foundations of cooperative preferences, and how they develop with age and differ across cultures and genders, is therefore an important topic. Experiments on children in different countries are one way to increase this understanding.

In this article we report on gender differences in cooperation among more than 800 children aged 9–12 years in Colombia and Sweden, two countries with clear differences regarding gender equality of opportunities and outcomes [20]. We introduce a novel version of a prisoner's dilemma that can be implemented in a Physical Education (PE) class. In this task, children are randomly paired, and decide in private how to divide a total of 10 balls between a private bin that gives three individual points for each ball and a public bin that gives two points per ball to each child. The balls are placed in the middle of the play area, and the bins are placed in pairs at each end, with each child having their own private bin and their own public bin. The children are then given two minutes to fetch the balls one by one and make their decisions on how to allocate the balls. This one-shot two player-game can thus be considered as a continuous prisoner's dilemma or a two-person public goods game. Even though there are ten allocation decisions, the children receive no feedback during the placement of the balls and the ten allocation decisions are thus combined into a single measure of cooperation which is simply the number of balls placed in the public bin.

We find no significant difference in the average level of cooperation between Colombia and Sweden overall. However, there is a significant difference in cooperation between girls from the two countries, where Colombian girls are less cooperative than Swedish girls. No differences in cooperation are found between boys in the two countries. Looking at gender differences within each country, girls in Colombia are in point estimates less cooperative than boys, whereas the point estimates are the opposite in Sweden. However, none of these differences are significant. We also find indications of the gender of the opponent to have an effect; children in our sample are more cooperative with boys than with girls. Importantly, even though we have a fairly large sample, the difference between Colombian and Swedish girls is the only result that survives a correction for multiple testing. In sum, our results suggest that it is important to avoid generalizing results from only one country when exploring gender differences in economic behaviors such as cooperation.

Earlier results on gender differences in cooperation in the prisoners' dilemma among adults are mixed, with some finding that men are more cooperative, some finding that women are more cooperative, and some finding no gender difference [4]. A few studies have set out to understand this variation further. For example, one study [5] explores the extent to which gender differences in cooperative behaviors are influenced by context, by having subjects in a prisoner's dilemma make their decision on whether to cooperate or defect while being observed by in-group members or out-group members. While men cooperate less when observed by their in-group compared to their out-group, women behave in the opposite way. Another study [6] uses a modified prisoner's dilemma to show that men in an all-male context are less likely to punish deviators from a cooperation norm compared both to men in a gender-mixed context and to women in either type of context. Together, these studies suggest that more attention should be paid to the contexts and to the gender-related social expectations in social dilemma games in order to systematically investigate gender differences in cooperation.

Among children, gender differences in cooperation remain largely unexplored; we are aware of only two studies in this area. One study finds no gender differences in a sample of American children aged 6–12 [7], whereas another shows that girls in another sample of American children are more cooperative than boys [8]. There are two other experiments that we are aware of on cooperation as measured by either a prisoner's dilemma or a public goods game among children, but these do not explore gender differences [9], [10].

In our study, the children get to know their counterpart just before the prisoner's dilemma game starts and cooperation is here likely to be coupled with indirect reciprocal behavior. According to evolutionary models, economic game theory and cross cultural studies, there are several tendencies among adults that promote cooperative behavior; to benefit close/kin relations, to directly reciprocate and to indirectly reciprocate through reputation, for example (see, e.g., [11], [12], [13], [14], [15]). Reciprocal behavior in the economic literature is often measured in connection to cooperative trust [16]. Among adults there is some evidence of men being more trusting and women more reciprocal [4]. The empirical literature on children is smaller. One study indicates that children as young as 3.5 years also exhibit these reciprocal tendencies in relation to cooperation [17], but they do not report on differences between genders. Unpublished data from Dalman, S, Ljungqvist, P and Johannesson, M. (http://www.econstor.eu/handle/10419/56345) show that reciprocal behavior increases with age. In this data gender does not seem to play a role. Further research investigates the development of trust and reciprocity in children but find no gender effect in share of reciprocal acts [18]. In line with this research on reciprocal responses to cooperative trust behavior among children indicate little or no effect of gender differences [19].

The present study aims to contribute to this relatively small body of literature by comparing the gender gap in cooperation among children in two countries that vary substantially in gender equality: Sweden, which typically places in the top of gender equality indices, and Colombia, which places substantially lower [20]. By using a controlled experiment replicated in both countries and with a large sample of about 800 children we expect to enhance the understanding of this area of research.

In an earlier article [21] we explored whether the gender gap in competitiveness and risk preferences differs systematically between Colombia and Sweden. Competitiveness in economics is typically measured by the change in performance when an individual performs under a tournament scheme relative to a piece-rate scheme, or by the individual's choice of being paid according to a tournament scheme or a piece-rate scheme. Risk preferences are typically measured by incentivized gambles. Given previous results on the gender gap in competitiveness across cultures and contexts, we expected boys to be more competitive than girls in Colombia, with a smaller gender gap — if any at all — in Sweden [22], [23]. However, we found no evidence of a gender gap in competitiveness in Colombia, whereas the results in Sweden were mixed depending on the task performed and the type of competitiveness measure. We also reported a larger gender gap in risk taking in Colombia compared to Sweden, with boys being more risk taking than girls in both countries. It is not obvious how these results should translate to gender gaps in cooperation in Colombia and Sweden, and previous literature on the topic is largely silent when it comes to making predictions about this. Thus, the current article remains largely explorative.

The outline of this article is as follows. We present the experimental setup in Section 2, we present our results in Section 3, and we finish with a discussion in Section 4.

## Methods

The experiment consisted of two parts. The first part was conducted during a Physical Education (PE) class and the second part was conducted in a classroom either on the same day or the same week. Both parts of the study were overseen by at least one teacher and two experimenters. The focus of the present article is the cooperation task, which was performed only in the PE class. In this part, the children participated in a cooperation task, and competed in running and skipping rope. The cooperation task was performed before the children were aware of the competitive elements of running and skipping rope. The other parts of the project concern competitiveness and the results are published in a previous paper [21]. Relevant for the analysis, we also measured risk preferences in the classroom. Risk preferences are here measured by the number of risky gambles chosen (the children were given several choices between various certain amounts and a 50-50 gamble, see [21]), with a higher number indicating more risk taking.

We invited all primary schools classes (grades 3–5) in Stockholm and Bogota to take part in the project. A total of 54 primary school classes participated. Whereas the children attending the participating schools may not constitute a representative sample of the respective national populations, the participating sample includes schools with diverse socioeconomic profile in both countries [see 21 for further description].

The cooperation task had the form of a Prisoner's Dilemma game in which each player made 10 subsequent allocation choices (this game can thus easily be transformed into a public goods game with more than two players). The units allocated were balls, and cooperation and defection were represented by two different physical bins, a private and a public bin, both placed within one, larger basket. The placement of the two smaller bins within the larger basket was only observed by the participating child. The two baskets were placed seven meters in opposite directions from a bag containing ten green balls and ten white balls. Children were randomly paired with an opponent (who was unknown until the task started), assigned a basket (with two separate bins) and a color. They were then given two minutes to fetch the 10 balls of the assigned color, one at a time, and place each of them either in the public or in the private bin. Each ball placed in the private bin gave three private points to that child only, whereas each ball in the public bin gave two points for each of the two children. The children were told that each ball was a choice and that they could place each ball in either of the two bins. If the class consisted of an odd number of children one child was randomly chosen to participate twice. In this case only the first participation of that child is used in the analysis. All children finished the task within the time limit of two minutes. The timing was introduced in order to make sure the children did not miss out on getting some exercise as part of the PE class.

Even though other children were present while the task was performed, measures were taken to ensure that the children made their choices in private. A child could for example not view the choices of their opponent. The total number of points earned was converted into attractive pens, markers and erasers and handed out in public at the end of the PE class. Each child received a bag with pens, markers and erasers proportional to the points earned but the individual points earned were never disclosed. The children were informed about the set-up at the beginning of the class, including the fact that more points would correspond to more prizes. A cooperation decision could be influenced by the fact that the children were aware of the setup and could potentially to some extent infer the behavior of the other child from the amount of prizes earned. Note however that this is kept constant across countries. Our measure of cooperation is the number of balls placed by each individual in her public bin.

### Ethics Statement

According to Swedish law, all research that comprises treatment of sensitive personal information, such as involving and handling of social security numbers, is subject to an ethical review by the Central (or Local) Ethical Review Board (Etikprövningsnämnden, EPN). Our research project did not handle social security numbers or other sensitive personal information, and did therefore not require review. We did not ask the Central Ethical Review Board for a written waiver. There has been no such praxis present in our field of study in Sweden. The decision in Sweden is and has been the discretion of the individual researcher and research departments. The Department of Economics at Stockholm University and the Department of Economics at Stockholm School of Economics did not consider the study being eligible for review or that it required collecting informed consent in line with the Swedish legislation on ethical review. The English document “The ethical Review act” provided by the Central Ethical Review Board contains a translation of paragraph 3 and 4 of the Swedish legislation concerning ethical review and consent. Paragraph 3 and 4 state what research is subject to this legislation (http://www.epn.se/media/45159/the_etical_review_act.pdf).

### Swedish legislation concerning ethical review of research involving humans: Lag (2003:460) om etikprövning av forskning som avser människor

**Forskning som omfattas av lagen. 3** § Denna lag ska tillämpas på forskning som innefattar behandling av.

1. känsliga personuppgifter enligt 13 § personuppgiftslagen (1998:204), eller
2. personuppgifter om lagöverträdelser som innefattar brott, domar i brottmål, straffprocessuella tvångsmedel eller administrativa frihetsberövanden enligt 21 § personuppgiftslagen. *Lag (2008:192)*.

**4 §** Utöver vad som följer av 3 § ska lagen tillämpas på forskning som

1. innebär ett fysiskt ingrepp på en forskningsperson,
2. utförs enligt en metod som syftar till att påverka forskningspersonen fysiskt eller psykiskt eller som innebär en uppenbar risk att skada forskningspersonen fysiskt eller psykiskt,
3. avser studier på biologiskt material som har tagits från en levande människa och kan härledas till denna människa,
4. innebär ett fysiskt ingrepp på en avliden människa, eller
5. avser studier på biologiskt material som har tagits för medicinskt ändamål från en avliden människa och kan härledas till denna människa. *Lag (2008:192)*.

English translation by the EPN:

**Research that is subject to the statute.** Section 3 This statute is applicable to research that involves dealing with

1. sensitive personal data as defined by section 13 of the Personal Data Act (1998:204), or
2. personal data concerning offences against the law that include crimes, judgments in criminal cases, coercive penal procedural measures or administrative deprivation of liberty as defined in section 21 of the Personal Data Act if the person who is the subject of the research has not expressly consented to this.

**Section 4** In addition to the consequences of section 3, this statute is to be applicable to research that

1. involves a physical intervention affecting a person who is participating in the research,
2. is conducted in accordance with a method intended to physically or mentally influence a person who is participating in the research,
3. concerns studies of biological material that has been taken from a living person and that can be traced back to that person,
4. involves a physical intervention upon a deceased person, or
5. concerns studies of biological material that has been taken for medical purposes from a deceased person and can be traced back to that person

In Colombia the Department of Economics at University of Los Andes did not consider the study being eligible for review by an Institutional Review Board (IRB), but required informed consent, in line with the decree from 1993 regulating ethical standards regarding research on health.

### Resolucion N° 008430 De 1993 (4 De Octubre De 1993)

***DISPOSICIONES GENERALES***

**ARTICULO 1.** Las disposiciones de estas normas científicas tienen por objeto establecer los requisitos para el desarrollo de la actividad investigativa en salud.

(We have omitted paragraph 2 and 3 here)

**ARTICULO 4.** La investigación para la salud comprende el desarrollo de acciones que contribuyan:

- Al conocimiento de los procesos biológicos y sicológicos en los seres humanos.
- Al conocimiento de los vínculos entre las causas de enfermedad, la práctica médica y la estructura social.
- A la prevención y control de los problemas de salud.
- Al conocimiento y evaluación de los efectos nocivos del ambiente en la salud.
- Al estudio de las técnicas y métodos que se recomienden o empleen para la prestación de servicios de salud.
- A la producción de insumos para la salud.

English translation (authors' translation):

**Paragraph 1.** The provisions of these scientific standards are intended to establish the requirements for the development of health research activity.

(We have omitted paragraph 2 and 3 here)

**Paragraph 4.** Health research includes the development of actions that contribute:

- To the knowledge of biological and psychological processes in humans.
- To the knowledge of the links between the causes of disease, medical practice and social structure.
- The prevention and control of health problems.
- To the knowledge and assessment of the harmful effects of the environment on health.
- The study of the techniques and methods that may be recommended or employed for the provision of health services.
- In the production of health products.

In both countries we first formally contacted the schools with an electronic letter explaining the scope and methodology of the study, and clarifying that no harm or risks were involved, physically or psychologically. All children would obtain a prize in the study. The teachers responsible were always present when we interacted with the children. The letter clarified that the activities we had designed in this study were equal to what the children would do during a regular school day, such as responding to mathematical and verbal questions and conducting PE exercises like running or skipping rope.

In Colombia we also asked the parents of the children in the schools and classes that agreed to participate to sign a written informed consent form and return it to the principal of the school. The written consent form asked for their consent of their children's participation and explained the features of the study, that no risk was involved for the participants, that the respect of privacy would be endorsed, and that the teacher would be present during the study. All children were allowed to participate, except one girl who suffered from asthma whose mother requested that she did not do the physical exercises.

## Results

In this section we test whether there is a gender gap in cooperation among children in Colombia and Sweden, within as well as between the countries. The analysis is based on how many units (balls) were placed in the public bin; the maximum possible number is 10 and the minimum is 0. We have performed Mann-Whitney tests as well as two-sided t-tests, but throughout the analysis we present only the p-value for the Mann-Whitney test unless the two tests vary in terms of significance, in which case both p-values are reported. Throughout the paper we consider a null-hypothesis to be rejected if the p-value is below 0.05. We present the Mann-Whitney test since none of our variables are normally distributed when using a skewness and kurtosis test. In order to test whether the size of the gender gap differs between Colombia and Sweden, we conduct a regression analysis.

### Basic statistics

A total of 1240 children (50% girls, 631 participants in Colombia and 609 in Sweden) predominantly aged 9–12 participated in the entire study during the academic year 2009–2010. Table 1 provides summary statistics pertaining to our sample in this paper, and Table A1 in File S1 provides variable descriptions. While there is likely some selection among the schools that participated, no self-selection occurred among the children since the participants comprised all children present in school on the day that the study took place. Among the participating classes, a subsample of classes was randomly chosen for the cooperation task, including 459 children in Colombia and 364 in Sweden.

**Table 1:** Summary statistics.

Variable	Mean	Sd	Median	N	Min	Max
**Age**	10.89	0.93	11	758	8	15†
**Gender (boy = 0, girl = 1)** *	0.51	0.50	1	808	0	1
**Country (Sweden = 1, Colombia = 0)**	0.44	0.50	0	823	0	1
**Contribution to PD**	4.11	4.16	3	823	0	10

* For 15 children we were not able to determine the gender.

† There is one child who is 15 years old, two who are 14 years old, 20 who are 13 years old, and three who are 8 years old. Age per se is however not the focus of the paper.

### Overall results

**Figure 1:**
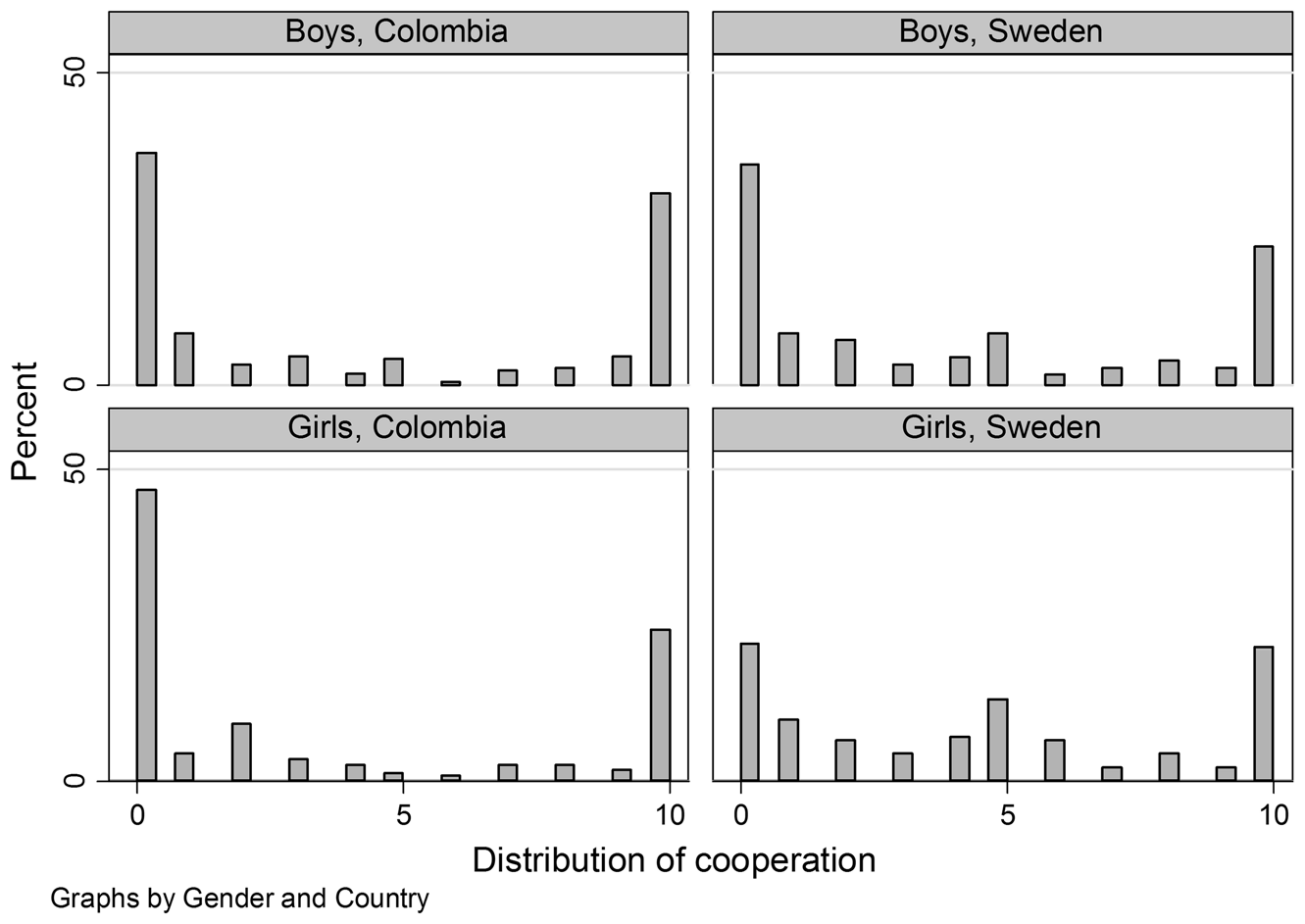
Distribution of cooperation, within gender and country. The bars represent the proportion of individuals, within the specific samples, that placed 0, 1, 2, 3, 4, 5, 6, 7, 8, 9, or 10 balls in the public bin.

Of the 10 units available the participating children allocate on average 4.11 units to the public bin. In point estimates, Colombian children cooperate somewhat less (4.0 units) than Swedish children (4.3 units). This difference is not significant using either the Mann-Whitney test, or the t-test (p (Mann-Whitney) = 0.0825, see Table 2, and p (t-test) = 0.3828), and we can thus not reject the null that there are no differences in cooperation between Colombia and Sweden. However, the Colombian distribution is more extreme than the Swedish one (Figure 1), with a larger proportion of children either cooperating fully or not at all. The test of equal distributions is rejected using a Kolmogorov-Smirnov test (p = 0.003).

**Table 2:** Average cooperation, null-hypothesis, and p-values.

Sample	Full sample	Boys sample	Girls sample	Null	p-value (MW)
**Full sample**	4.11	4.24	3.99	H_0_: B = G**	0.5911
**Colombian sample**	4.00	4.46	3.54	H_0_: B = G	0.0291
**Swedish sample**	4.25	3.98	4.55	H_0_: B = G	0.0664
**Null**	H_0_: C = S*	H_0_: C = S	H_0_: C = S		
**p-value (MW)**	0.0825	0.4426	0.0006		

* H_0_: C = S refers to the null-hypothesis of no differences in cooperation between Colombia (C) and Sweden (S) with the respective samples.

** H_0_: B = G refers to the null-hypothesis of no differences in cooperation between Boys (B) and Girls (G) with the respective samples.

### Gender differences within countries

We find no significant difference in average cooperation between boys (4.2 units) and girls (4.0 units) (p = 0.5911). However, in Colombia girls cooperate significantly less than boys (p = 0.0291). In Sweden, we only find that girls in point estimates cooperate more than boys (p(Mann-Whitney) = 0.0664; p(t-test) = 0.164). Table 2 displays average cooperation for each country by gender. We further find no distributional differences between the genders, within the sample as a whole or within each country respectively. See Graph 1.

### Gender differences between countries

Comparing the difference in how children behave across countries, we find some indication of difference between Colombia and Sweden. This difference is driven by the gap in cooperation between Colombian and Swedish girls. As can be seen in Table 2, Colombian girls cooperate the least and Swedish girls the most, and these groups differ significantly (p = 0.0006). Boys in the two countries behave similarly (p = 0.4426). To probe further into this result we compare the distribution of girls in the two different countries. The distribution among Colombian girls is more extreme, with a larger share of Colombian than Swedish girls putting 0 balls in the public bin. A Kolmogorov-Smirnov test also indicates different distributions among girls across countries (p<0.001), but not among boys (p = 0.229). Graph 1 displays the distribution of boys and girls within the respective countries.

### Gender of the opponent

Previous literature has also explored whether behavior is affected by the gender of the counterpart (see [4] for a review). We find that in the full sample, average cooperation when a child faces a boy compared to a girl is 4.45 and 3.80 units respectively; a significant difference in cooperation (p = 0.0125). Separating the sample by gender, the effect is significant among girls (p = 0.0395). For boys, there is some evidence of an effect (p = 0.0837), however this is not significant with a t-test (p = 0.219). Analyzing boys and girls in each country separately, there is little evidence that the gender of the opponent matters in any group (see Table 3).

**Table 3:** Average cooperation based on gender of opponent.*

Sample	N		Gender of matched counterpart			
		Boys	Girls	Difference	Null**	p-value
**Colombia**	219/239	4.41	3.63	0.78	H_0_: MB = MG	0.0543
**Boys vs.**	115/103	4.69	4.20	0.49	H_0_: MB = MG	0.2089
**Girls vs.**	99/127	4.17	3.06	1.11	H_0_: MB = MG	0.1416
**Sweden**	182/179	4.51	4.03	0.48	H_0_: MB = MG	0.1374
**Boys vs.**	82/97	4.30	3.78	0.52	H_0_: MB = MG	0.2331
**Girls vs.**	100/81	4.67	4.38	0.29	H_0_: MB = MG	0.5128

The rows indicate within which sample the test is conducted, and the third and fourth columns indicate the gender of the matched counterpart.

*We lack information about the gender of the opponent for four participants.

** H_0_: B = G refers to the null-hypothesis of no differences in cooperation between being randomly matched with a Boy (MB) and being randomly matched with Girl (MG) with the respective samples.

### Robustness

In this study we conduct multiple tests, something that may increase the probability of Type I errors. We did not, however, take Bonferroni corrections into account when designing the study, and do not include the corrections in the text partly in order to avoid an increase of Type II errors (see power analysis below). To provide a more cautious interpretion of the results we therefore also look at the results using Bonferroni correction for multiple comparisons. Here we take all 12 tests we conduct into account (dependent as well as independent); 6 test studying differences in general cooperation and 6 tests looking at gender of the opponent. The p-value required for a significant result is then 0.05/12 = 0.004. A similar adjustment of the significance level would imply that our only remaining statistically significant result is the difference between Colombian girls and Swedish girls (p = 0.0006).

In order to further assess the stability of our results we conduct a power analysis for each significant test result and a sample size analysis for all null-results. The powers of the tests are all below 0.8 which is a commonly used standard. However, the difference between girls in Colombia and Sweden has a power of 0.7250. This analysis provides a similar picture as the Bonferroni corrections. See Table A3 in File S1.

We also compare the main results from regressions with no control variables included with regressions controlling for age as well as risk preferences, using both ordinary least squares and Tobit regressions (lower limit = 0 and upper limit = 10). The regressions give marginally significant p-values for the interaction between country and gender, which remain when controlling for age and risk preferences in the analysis using Tobit but disappears when we use ordinary least squares. The differences in results between the Tobit and ordinary least squares regressions could be caused by randomness or by differences in distributions since we have a truncated dependent variable, something that only Tobit addresses. The coefficients, significance and R^2^ (or pseudo R^2^) are presented in Table A2 in File S1. We also find that age correlates positively and significantly with cooperation such that older children are more cooperative. Throughout, our results are qualitatively similar when we also control for school affiliation. As reported in our previous study [21], there are also gender differences in risk taking in the pooled data (p<0.001). However, we do not find any evidence of a significant correlation between risk preferences and cooperation.

## Discussion

In this paper we have introduced a new measure of cooperation. This measure illustrates the dynamics of the prisoners' dilemma and the public goods game; it is also easily understood, and therefore suitable for running experiments on children. Moreover, this measure can be implemented during a PE class and does not require elaborate resources, and is therefore easy to use in a wide range of settings and with different age groups. In particular, it is useful in studies like the one described here, covering two different countries. This explorative study compares children from quite different societies in terms of culture (including gender norms and gender equality in access to opportunities and outcomes) and socio-economic backgrounds [20]. Moreover, the cooperation task involves both a physical component associated with the effort of running to collect the balls and the decision whether to cooperate or not. The combination of effort and payoff structure provides a realistic task which illustrates the prisoner's dilemma for children. However, our study does not include repeated measures of cooperative behavior. This may be a limitation to the external validity of the study and calls for an extension of this experimental design. The gender-specific differences we find may increase or decrease with repeated interaction. Little is known, however, about gender differences in cooperation among children. Our results show that Swedish girls cooperate more than Colombian girls. Coupled with this we find indications that girls are less cooperative than boys in Colombia whereas no gender differences are found in Sweden. This result should however be interpreted with caution since it has low power and would not be significant using Bonferroni corrections. Further, since children know the identity of their counterpart when the game starts, cooperation can here be coupled with reciprocal preferences. In sum, our results indicate that differences in cooperation among children may differ across countries and be gender specific. It is thus important to systematically study behavior in different countries or contexts in order to draw general conclusions about gender differences. This general result is also supported by a previous paper [24] that finds higher cooperation among adults in a matrilineal society compared to adults in two patriarchal societies in India. Interestingly, and in contrast to our results among children, this difference across societies among adults is mainly due to a difference in how men behave.

We are unaware of other studies exploring gender differences in cooperation among children in different countries. However, there is one relevant study on social preferences measured from modified dictator games in a sample of Swedish and Austrian children aged 10–15 years old [25]. Boys are found to be more efficiency concerned and girls more inequality averse, and Swedish children to be more social-welfare oriented and less difference averse than Austrian children. A natural extension of the work described in this article would be to use our cooperation setup and also measure social preferences.

Exploring behavior in different contexts is important for understanding the determinants of the gender gap in preferences. Our experimental design could be easily used as a monitoring device for evaluating interventions aimed at diminishing these gender gaps. Moreover, studies on children can increase our understanding of how preferences develop over age, and how children's behavior compares to that of adults. Future research should expand this type of work by comparing different age groups and countries, while at the same time systematically varying key features of the context in order to understand the determinants of a wide range of preferences.

## Supporting Information

File S1 **Supporting Information.** Table A1. Set of variables used, variable description. Table A2. Cooperation regressions. Table A3. Power tests and sample size tests.
(DOCX)
