# Supplementary material for: Gender and Cooperation in Children: Experiments in Colombia and Sweden
Source: PLoS One. 2014 Mar 10;9(3):e90923. doi: 10.1371/journal.pone.0090923 (PMC3948702; doi:10.1371/journal.pone.0090923)
Supplement: File S1 — Supporting Information. Table A1. Set of variables used, variable description. Table A2. Cooperation regressions. Table A3. Power tests and sample size tests. (DOCX) [file pone.0090923.s001.docx]

**Supporting Information S1**

**Table A1. Set of variables used, variable description**

| **Sweden** (Colombia=0, Sweden=1) | Dummy variable for country |
| --- | --- |
| **Female**(Boy=0, Girl=1) | Dummy variable for gender |
| **Female*Sweden** | Interaction variable between gender and country |
| **Cooperation** | Continuous measure of cooperation |
| **Age** | Age measured in years |
| **Risk** | Number of risky choices* |

***** The risk measure consisted of six lotteries where the children could choose between a coin flip that gives 10 or 0 points with equal probability and a certain amount that increases successively in points (from 2 to 7.5 points). We measure risk by the number of times a person chooses the uncertain option. The observations on risk preferences have previously been reported in Cárdenas et al 2012.

**Table A2. Cooperation regressions**

| **VARIABLES** | **OLS** | **Tobit** | **OLS with controls** | **Tobit with controls**^§^ |
| --- | --- | --- | --- | --- |
| **Female** | -0.642 | -1.527 | -0.565 | -1.358 |
|  | (0.440) | (1.086) | (0.449) | (1.102) |
| **Sweden** | -0.108 | 0.0405 | -0.173 | -0.0935 |
|  | (0.453) | (1.067) | (0.450) | (1.060) |
| **Female*Sweden** | 1.059* | 2.678* | 0.979 | 2.472* |
|  | (0.613) | (1.449) | (0.612) | (1.443) |
| **Age** |  |  | 0.401** | 1.102*** |
|  |  |  | (0.170) | (0.406) |
| **Risk** |  |  | 0.0407 | 0.0457 |
|  |  |  | (0.0983) | (0.245) |
| **Constant** | 4.194*** | 3.250*** | -0.278 | -8.867* |
|  | (0.323) | (0.774) | (1.907) | (4.584) |
| **Observations** | 712 | 712 | 712 | 712 |
| **R^2^/Pseudo R^2^** | 0.007 | 0.003 | 0.016 | 0.005 |

Standard errors in parentheses *** p<0.01, ** p<0.05, * p<0.1
§ We here use the lower limit set to 0 and the upper limit set to 10

**Table A3. Power tests and sample size tests**

| Sample | Full sample |  | Colombia | Sweden | Men | Women |
| --- | --- | --- | --- | --- | --- | --- |
| Test | **Between genders** | **Between countries** | **Between genders** | **Between genders** | **Between countries** | **Between countries** |
| Power |  |  | 0.6093 |  |  | 0.7250 |
|  |  |  |  |  |  |  |
| Actual sample size | 808 | 823 | 445 | 363 | 399 | 409 |
| Required sample size | 11415 | 11073 | - | 1966 | 3287 | - |
| Diff (required-actual) | 10607 | 10250 | - | 1603 | 2888 | - |

* The sample size and power tests are based on the tests provided in Table 2. Average cooperation.
** In the case of a significant test we have conducted a power analysis. The respective required sample sizes stated are a sum of the required sample for each sub-group of the respective tests.
